# Strangulated cholecystitis in an umbilical hernia: world-first case report

**DOI:** 10.3389/fmed.2025.1684301

**Published:** 2026-01-09

**Authors:** Marco Brolese, Matteo Pittacolo, Arianna Vittori, Gianpietro Zanchettin, Renato Salvador, Valeria Valli, Lorenzo Vallese, Nicola Baldan, Gianfranco Da Dalt, Michele Valmasoni, Alberto Friziero

**Affiliations:** Acute Care Surgery Unit, Department of Surgery, Oncology and Gastroenterology, University of Padova, Azienda Ospedale Università Padova, Padova, Italy

**Keywords:** acute care surgery, acute cholecystitis, case reports, gallbladder diseases, umbilical hernia

## Abstract

**Background:**

Strangulated umbilical hernia and acute cholecystitis are among the most common surgical emergencies, with operative management remaining the cornerstone of treatment. This study describes, to our knowledge, the first documented case worldwide of acute cholecystitis strangulated within an umbilical hernia.

**Case presentation:**

A 97-year-old woman with a history of recurrent choledocholithiasis presented to the Acute Care Department with abdominal pain localized to the mesogastrium and an irreducible, tender periumbilical mass. Abdominal computed tomography scan described a strangulated intestinal loop, prompting urgent surgical intervention. Surprisingly, exploratory laparotomy revealed an acute cholecystitis strangulated within the umbilical hernia, along with marked dilation of the common bile duct measuring up to 2 cm. Thus, a cholecystectomy and common bile duct exploration were performed, revealing a 2 cm non-obstructive gallstone at the ampulla of Vater, which was successfully extracted. A T-tube was then placed for biliary drainage, and the abdominal wall was closed primarily. The patient was discharged on postoperative day 14 in fair overall clinical condition and the subsequent follow-up was regular.

**Conclusion:**

This case underscores the importance of maintaining a high index of suspicion for atypical hernia contents and highlights the need for individualized surgical decision-making. Gallbladder herniation, though rare, can lead to diagnostic challenges. Successful management requires both technical expertise and sound clinical judgment in urgent settings.

## Background

Strangulated umbilical hernia and acute cholecystitis are among the most frequent surgical emergencies. Umbilical hernias in adults represent approximately 6 to 14% of all abdominal wall hernias, while acute cholecystitis accounts for an estimated 200,000 diagnoses annually in the United States (1, 2). Strangulation can result in bowel obstruction and, in severe cases, may progress to strangulation and visceral ischemia, requiring urgent surgical intervention, as recommended by the World Society of Emergency Surgery (WSES) (3). Cholecystitis is one of the most common complications of cholelithiasis, presenting as the initial clinical manifestation in 10–15% of cases, and accounting for 3 to 9% of all hospital admissions for acute abdomen in adults (4). Additionally, choledocholithiasis occurred in 5–15% of these patients, and it significantly increases the risk of other severe complications, such as acute cholangitis and biliary pancreatitis (5). Surgical intervention remains the cornerstone of treatment for both acute cholecystitis and strangulated umbilical hernia (6, 7). Although these conditions are well recognized independently, emergency surgery often presents unpredictable scenarios, where timely diagnosis and appropriate management are critical in determining patient outcomes. In this case report, we describe an exceptionally rare presentation of acute cholecystitis strangulated within an umbilical hernia, a combination which, to our knowledge, has not been previously described in the literature. This case underscores the importance of adequate surgical expertise when managing such complex and atypical clinical scenarios, as well as the need for heightened awareness of rare, but potentially life-threatening, presentations of common surgical diseases.

## Case presentation

**Figure 1:**
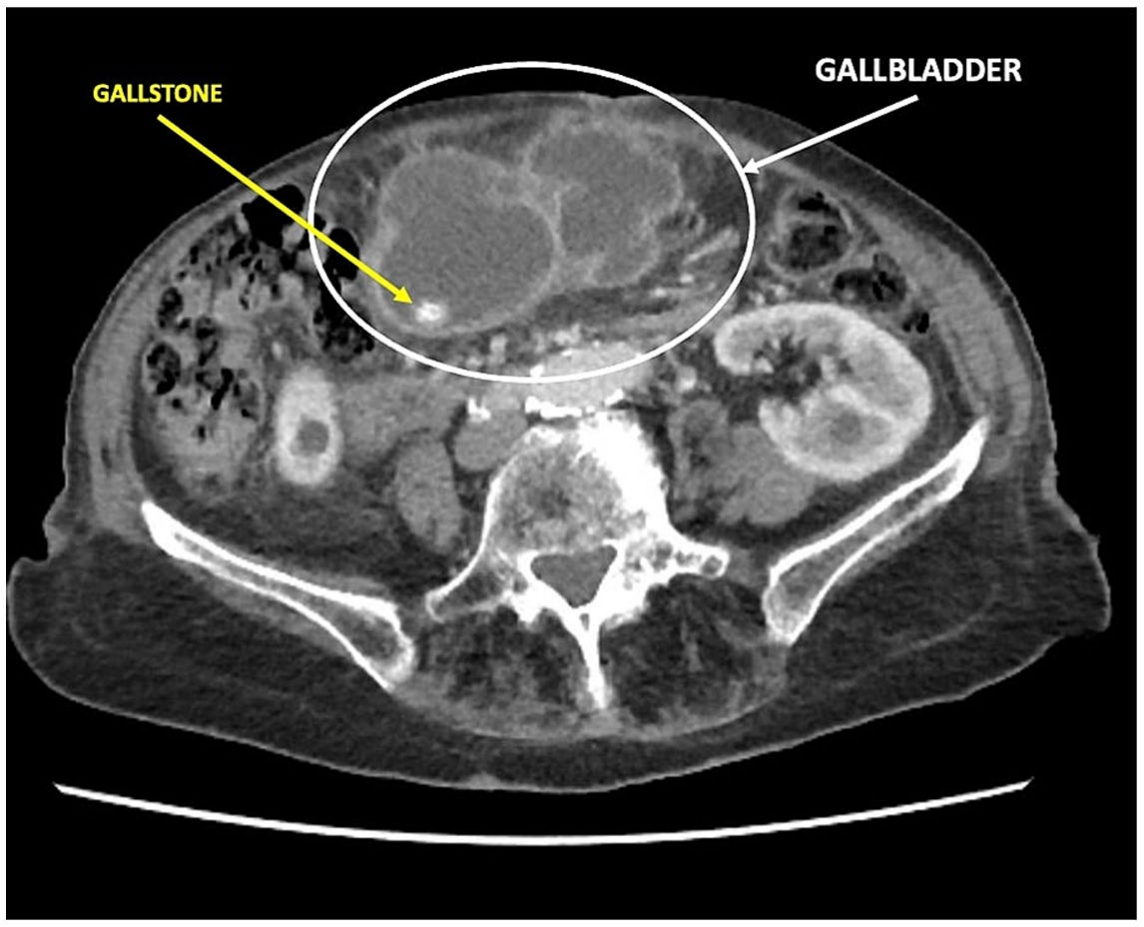
CT scan during first admission showed a markedly distended gallbladder extending into the mesogastric region, without hernial orifice involvement.

**Figure 2:**
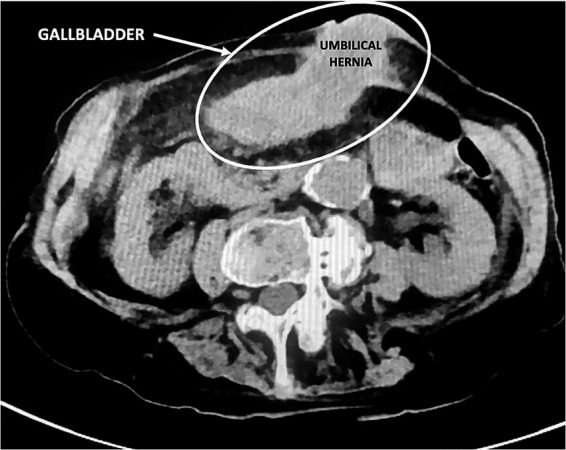
Preoperative CT scan clearly revealed what was later identified as the gallbladder within the umbilical hernia.

**Figure 3:**
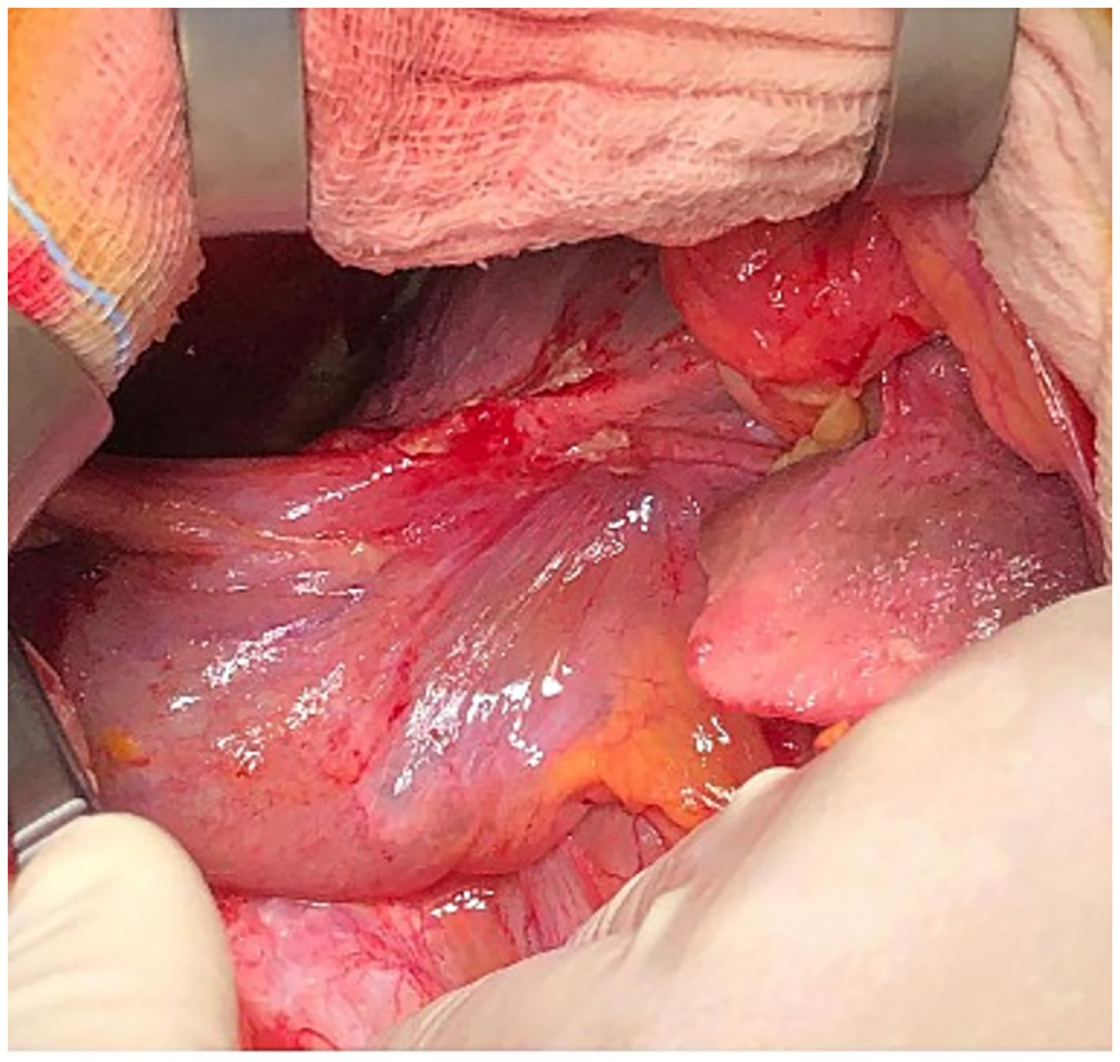
Intraoperative picture showing the gallbladder having fragile and inflamed walls.

**Figure 4:**
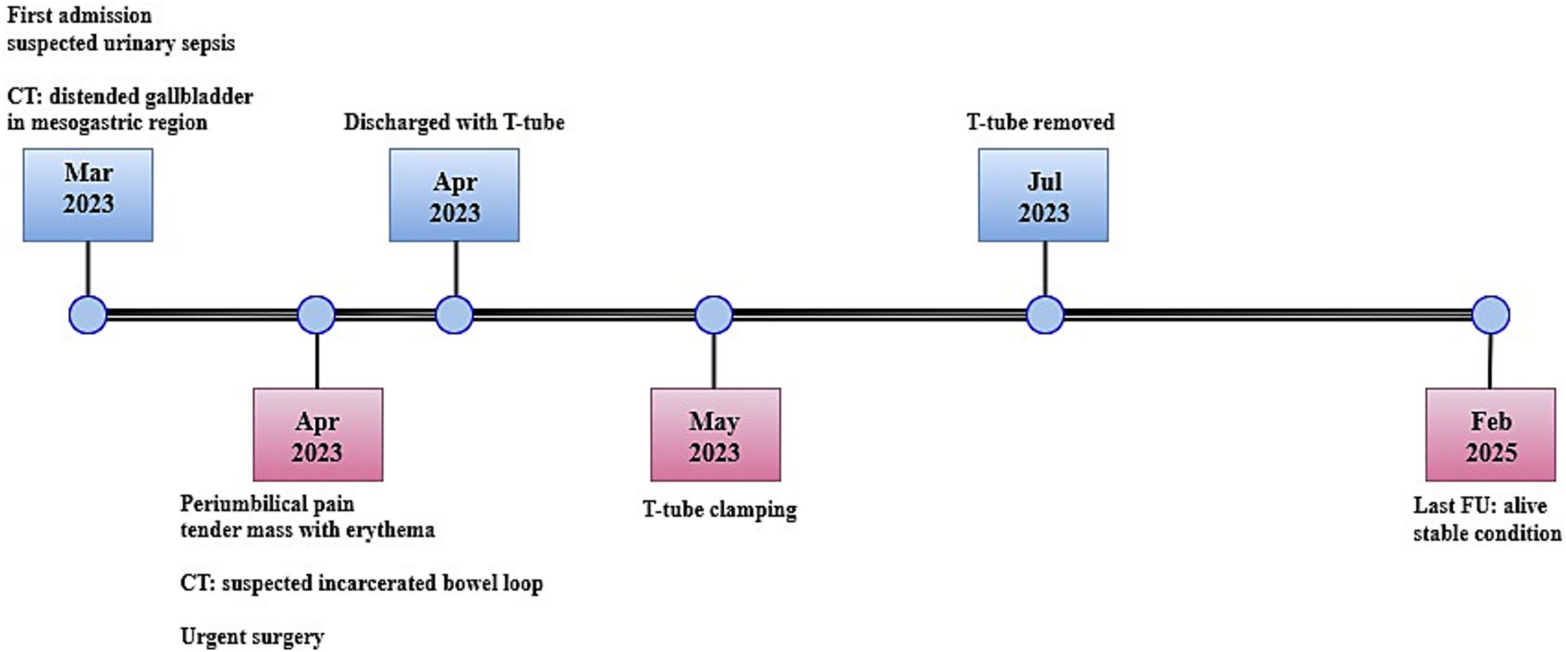
Timeline.

In March 2023, a 97-year-old woman was hospitalized for 20 days in the General Medicine Department of our Hospital due to suspected urinary sepsis, supported by positive urinalysis and urine culture findings. Despite her advanced age, the patient was in fair overall clinical condition. She was 151 cm tall, weighed 47 kg (body mass index 20.6 kg/m^2^), and had a medical history of hypertension, osteoporosis, bronchial asthma, and asymptomatic umbilical hernia. The patient also had a documented history of cholecysto-choledocholithiasis; however, no prior surgical or endoscopic treatments were documented, and detailed information regarding previous management was unavailable. During her hospital stay, she was successfully managed conservatively with antibiotic therapy. However, given the presence of initial nonspecific abdominal symptoms, a computed tomography (CT) scan was also performed. Findings excluded the presence of surgical emergencies, with the only abnormality being a distended gallbladder positioned in the mesogastric region, with mildly thickened walls (Figure 1). However, on clinical examination Murphy and Blumberg signs were negative. Despite radiological suspicion of mild cholecystitis, her advanced age and post-antibiotic clinical improvement led to her being deemed clinically stable and discharged. Two weeks later, the patient presented to the Acute Care Department with new-onset abdominal pain in the periumbilical region. On clinical examination, a tender mass approximately 4 cm in diameter was noted in the umbilical area, with visible redness of the overlying skin. Laboratory tests showed neutrophilic leukocytosis (13.7 × 10^9^/L) and elevated C-reactive protein (103 mg/L). Hemoglobin was within the normal range (12.4 g/dL), as were total bilirubin (0.8 mg/dL) and liver enzymes, including transaminases, alkaline phosphatase, and gamma-glutamyl transferase. A mild renal impairment was also noted, with serum creatinine slightly above the normal range (1.3 mg/dL), and blood urea modestly elevated (7.9 mmol/L). Lactate levels were slightly elevated at 2.4 mmol/L. Due to the clinical presentation and laboratory findings a contrast-free CT scan was performed (Figure 2). The radiological report described a strangulated bowel loop within a 3-cm umbilical orifice. Nevertheless, the gallbladder enlargement noted on the prior CT scan left open the possibility of its involvement. Urgent surgical intervention under general anesthesia was therefore planned. A lower midline laparotomy, far from the hernia site, was initially performed to avoid visceral injuries. The incision was then extended cranially. Intraoperative findings excluded bowel ischemia, while revealing the presence of acute cholecystitis (Figure 3): a huge gallbladder was found twisted along its longitudinal axis, with inflamed and fragile but intact walls, including the fundus, which was strangulated within the hernial orifice. The hernial neck was resected first to avoid gallbladder rupture and prevent contamination of the operative field. Following careful dissection, an anterograde cholecystectomy was carried out. Furthermore, given the abnormal dilation of the common bile duct (2 cm) and the patient’s known history of cholecysto-choledocholithiasis, a longitudinal choledochotomy was also performed. Intraoperative choledochoscopy using a flexible choledochoscope revealed a large 2 cm gallstone partially obstructing the distal common bile duct, in close proximity to the ampulla of Vater. The stone was successfully extracted using forceps, and a T-tube was subsequently placed for biliary drainage. Finally, hernia repair was completed with primary abdominal wall closure. The postoperative course was complicated by the onset of pneumonia on postoperative day 4, which was successfully treated with a penicillin. A bile leak was also revealed by the subhepatic surgical drain. The patient was discharged on postoperative day 14 in fair overall clinical condition, with the T-tube left open, draining approximately 200 cc per day, while the surgical drain output was around 50 cc per day. After discharge, the bile leak gradually decreased, leading to the T-tube being clamped 1 month later. Subsequently, the surgical drain was removed, while the T-tube was taken out 3 months after surgery without complications, following a control cholangiography. Histological examination revealed acute empyematous cholecystitis. The patient was subsequently evaluated in the outpatient clinic every 6 months until the last follow-up in February 2025, at which time she was alive and in a stable overall condition. Figure 4 show the case presentation timeline.

## Discussion

This is the first reported case of acute cholecystitis within a strangulated umbilical hernia. While gallbladder herniation itself is extremely rare, recent evidence suggests that the coexistence of cholelithiasis and abdominal wall hernias is not uncommon. In a prospective multicenter study, Kulacoglu et al. (8) reported that 25.9% of patients with gallstones had an umbilical hernia and 29.6% of umbilical hernia patients had cholelithiasis, indicating a high prevalence of co-occurrence. This supports the notion that, although herniation of the gallbladder is rare, clinicians should be aware of the frequent association between gallstones and hernias.

Rarely, the gallbladder protrudes into the inguinal canal or through incisional hernias, and is often identified incidentally or in the context of complications, such as acute calculous cholecystitis (9, 10). As well known, strangulated hernias are in general associated with high morbidity and mortality, particularly when complicated by strangulation or necrosis. The presence of necrosis is the strongest independent predictor of adverse outcomes, while advanced age, comorbidities, delayed presentation, and high American Society of Anesthesiologists score further increased risk (11, 12). In our case, the underlying chronic choledocholithiasis, further supported by the absence of both intrahepatic bile duct dilation and jaundice, played a significant role in the development of this atypical scenario. In fact, the chronic partial obstruction of the common bile duct likely caused progressive enlargement of both the common bile duct and, predominantly, the gallbladder which, coupled with the patient’s small habitus, facilitated the displacement of the gallbladder through the umbilical orifice. This led to strangulation of the gallbladder, further intensifying the inflammatory response, which was ultimately driven by multiple contributing factors. The decision to surgically explore the common bile duct has represented a critical step. Kaim et al. reported an average common bile duct diameter of 6.5 ± 2.5 mm (13) in the elderly population, however, in our case, a diameter of 2 cm was found, strongly suggestive of choledocholithiasis despite normal bilirubin levels. According to several guidelines, in patients with both acute cholecystitis and choledocholithiasis, the recommended approach typically involves endoscopic retrograde cholangiopancreatography (ERCP), either pre- or post-cholecystectomy, or intraoperatively (14, 15). Nonetheless, in our patient, the clinical priority was to address the suspected bowel ischemia, initially raised by the radiologist’s interpretation, and further supported by abdominal examination. Performing a simultaneous surgical-endoscopic rendez-vous in the operating room is not always feasible due to technical or logistical constraints, and deferring the endoscopic procedure after surgery was not considered a safe option in this situation. If postoperative ERCP had been undertaken, the absence of the gallbladder as a reservoir would likely have led to biliary hypertension, heightening the risk of bile leakage from the cystic duct stump. Moreover, due to the gallstone size, endoscopic extraction would have been technically unfeasible, further justifying the need for intraoperative biliary exploration. Surgical choledochoscopy was therefore deemed mandatory. Notably, this technique requires a high level of surgical expertise, highlighting the importance of biliary complication management as a fundamental skill in emergency surgery. Although the literature reports comparable outcomes between T-tube placement and primary closure, in this case, considering the extensive choledochotomy required for the large stone extraction, and the patient’s advanced age, we opted for the more conservative approach of T-tube drainage (16). Timely intervention prevented gallbladder perforation and subsequent development of bile peritonitis, a condition associated with morbidity and mortality rates of 57.7 and 9.5%, respectively (17). Although current guidelines typically recommend the placement of a preperitoneal flat mesh for defects greater than 1 cm with uncontaminated surgical field, we opted for direct closure considering the patient’s age and the inherent risk of bile leakage following T-tube placement (7). This case offers meaningful reflections on therapeutic decision-making in a complex surgical scenario. It is important to recognize that gallbladder herniation is an exceedingly rare occurrence, yet when it does occur, it can lead to diagnostic pitfalls. Clinical guidelines must be adapted and carefully tailored to the patient, allowing for the most appropriate therapeutic strategy based on the specific clinical context. This experience highlights how the acute care surgeon must be equipped not only with technical skills, but also with the capacity to make high-stakes decisions in uncertain and rapidly changing situations.

### Patient perspective

The patient expressed great satisfaction with the care received, highlighting the promptness of the intervention and the successful resolution of such a complex case.

## Data Availability

The original contributions presented in the study are included in the article/Supplementary material, further inquiries can be directed to the corresponding author.
